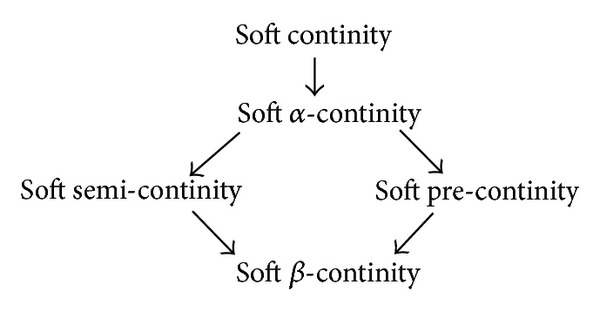# On Soft *β*-Open Sets and Soft *β*-Continuous Functions

**DOI:** 10.1155/2014/843456

**Published:** 2014-06-17

**Authors:** Metin Akdag, Alkan Ozkan

**Affiliations:** Department of Mathematics, Science Faculty, Cumhuriyet University, Sivas, Turkey

## Abstract

We introduce the concepts soft *β*-interior and soft *β*-closure of a soft set in soft topological spaces. We also study soft *β*-continuous functions and discuss their relations with soft continuous and other weaker forms of soft continuous functions.

## 1. Introduction and Preliminary

The concept of soft sets was first introduced by Molodtsov [1] in 1999 who began to develop the basics of corresponding theory as a new approach to modeling uncertainties. In [1, 2], Molodtsov successfully applied the soft theory in several directions such as smoothness of functions, game theory, operations research, Riemann integration, Perron integration, probability, and theory of measurement.

In recent years, an increasing number of papers have been written about soft sets theory and its applications in various fields [3, 4]. Shabir and Naz [5] introduced the notion of soft topological spaces which are defined to be over an initial universe with a fixed set of parameters. In addition, Maji et al. [6] proposed several operations on soft sets, and some basic properties of these operations have been revealed so far.

In general topology, the concept of *β*-open sets was introduced in [7] and *β*-open sets have been referred to as semipreopen by Andrijevic [8]. In [9] this concept has been generalized to soft setting. Our motivation in this paper is to define soft *β*-interiors and soft *β*-closures and investigate their properties which are important for further research on soft topology. These researches not only can form the theoretical basis for further applications of topology on soft sets but also lead to the development of information system and various fields in engineering. Furthermore, we will study soft *β*-continuous functions and obtain some characterizations of such functions.

**Definition 1 (see [1]).**

Let *X* be an initial universe and let *E* be a set of parameters. Let *P*(*X*) denote the power set of *X* and let *A* be a nonempty subset of *E*. A pair (*F*, *A*) is called a soft set over *X*, where *F* is a mapping given by *F* : *A* → *P*(*X*). In other words, a soft set over *X* is a parameterized family of subsets of the universe *X*. For *ε* ∈ *A*, *F*(*ε*) may be considered as the set of *ε*-approximate elements of the soft set (*F*, *A*).

**Definition 2 (see [6]).**

A soft set (*F*, *A*) over *X* is called a null soft set, denoted by Φ, if *e* ∈ *A*, *F*(*e*) = *∅*.

**Definition 3 (see [6]).**

A soft set (*F*, *A*) over *X* is called an absolute soft set, denoted by $\tilde{A}$, if *e* ∈ *A*, *F*(*e*) = *X*.

If *A* = *E*, then the *A*-universal soft set is called a universal soft set, denoted by $\tilde{X}$.

**Definition 4 (see [5]).**

Let *Y* be a nonempty subset of *X*; then $\tilde{Y}$ denotes the soft set (*Y*, *E*) over *X* for which *Y*(*e*) = *Y*, for all *e* ∈ *E*.

**Definition 5 (see [6]).**

The union of two soft sets of (*F*, *A*) and (*G*, *B*) over the common universe *X* is the soft set (*H*, *C*), where *C* = *A* ∪ *B* and for all *e* ∈ *C*,

(1) $$\begin{array}{c} H\left(e\right)=\{\begin{array}{cc} F\left(e\right), & \text{if}\;e\in A-B,\\ G\left(e\right), & \text{if}\;e\in B-A,\\ F\left(e\right)\cup G\left(e\right), & \text{if}\;e\in A\cap B. \end{array} \end{array}$$

We write $\left(F,A)\tilde{\cup }(G,B)=(H,C\right)$.

**Definition 6 (see [6]).**

The intersection (*H*, *C*) of two soft sets (*F*, *A*) and (*G*, *B*) over a common universe *X*, denoted by $\left(F,A)\tilde{\cap }(G,B\right)$, is defined as *C* = *A*∩*B*, and *H*(*e*) = *F*(*e*)∩*G*(*e*) for all *e* ∈ *C*.

**Definition 7 (see [6]).**

Let (*F*, *A*) and (*G*, *B*) be two soft sets over a common universe *X*. $\left(F,A)\tilde{\subset }(G,B\right)$, if *A* ⊂ *B*, and *H*(*e*) = *F*(*e*) ⊂ *G*(*e*) for all *e* ∈ *A*.

**Definition 8 (see [5]).**

Let *τ* be the collection of soft sets over *X*; then *τ* is said to be a soft topology on *X* if it satisfies the following axioms:

1. $\Phi ,\tilde{X}$ belong to *τ*,
2. the union of any number of soft sets in *τ* belongs to *τ*,
3. the intersection of any two soft sets in *τ* belongs to *τ*.

The triplet (*X*, *τ*, *E*) is called a soft topological space over *X*. Let (*X*, *τ*, *E*) be a soft topological space over *X*; then the members of *τ* are said to be soft open sets in *X*. The relative complement of a soft set (*F*, *A*) is denoted by (*F*,*A*)^*c*^ and is defined by (*F*,*A*)^*c*^ = (*F* ^*c*^, *A*) where *F* ^*c*^ : *A* → *P*(*X*) is a mapping given by *F* ^*c*^(*e*) = *X* − *F*(*e*) for all *e* ∈ *A*. Let (*X*, *τ*, *E*) be a soft topological space over *X*. A soft set (*F*, *A*) over *X* is said to be a soft closed set in *X*, if its relative complement (*F*, *A*)^*c*^ belongs to *τ*. If (*X*, *τ*, *E*) is a soft topological space with $\tau =\{\Phi ,\tilde{X}\}$, then *τ* is called the soft indiscrete topology on *X* and (*X*, *τ*, *E*) is said to be a soft indiscrete topological space. If (*X*, *τ*, *E*) is a soft topological space with *τ* being the collection of all soft sets which can be defined over *X*, then *τ* is called the soft discrete topology on *X* and (*X*, *τ*, *E*) is said to be a soft discrete topological space.

**Definition 9 .**

Let (*X*, *τ*, *E*) be a soft topological space over *X* and let (*F*, *A*) be a soft set over *X*.

1. Reference [4]: the soft interior of (*F*, *A*) is the soft set $\mathrm{int}\left(\left(F,A\right)\right)=\cup \left\{\left(O,A\right):\left(O,A\right)\;\text{is}\;\text{soft}\;\text{open}\;\text{and}\;(O,A)\tilde{\subset }(F,A)\right\}$.
2. Reference [5]: the soft closure of (*F*, *A*) is the soft set $\mathrm{cl}((F,A))=\cap \left\{\left(F,E\right):\left(F,E\right)\;\text{is}\;\text{soft}\;\text{closed}\;\text{and}\;(F,A)\tilde{\subset }(F,E)\right\}$.

Clearly cl⁡((*F*, *A*)) is the smallest soft closed set over *X* which contains (*F*, *A*) and int⁡((*F*, *A*)) is the largest soft open set over *X* which is contained in (*F*, *A*).

**Definition 10 .**

A soft set (*F*, *A*) of a soft topological space (*X*, *τ*, *E*) is said to be

- soft open [5] if its complement is soft closed,
- soft *α*-open [10] if $\left(F,A)\tilde{\subset }\mathrm{int}(\mathrm{cl}(\mathrm{int}((F,A)))\right)$,
- soft preopen [9] if $\left(F,A)\tilde{\subset }\mathrm{int}(\mathrm{cl}((F,A))\right)$,
- soft semiopen [11] if $\left(F,A)\tilde{\subset }\mathrm{cl}(\mathrm{int}((F,A))\right)$,
- soft *β*-open [9] if $\left(F,A)\tilde{\subset }\mathrm{cl}(\mathrm{int}(\mathrm{cl}((F,A)))\right)$.

**Proposition 11 .**

(a) Every soft open set is soft *α*-closed. (b) Every soft *α*-open set is soft preopen. (c) Every soft *α*-open set is soft semiopen. (d) Every soft semiopen set is soft *β*-open. (e) Every soft preclosed set is soft *β*-open.

**Proof**

The proof is obvious from Definition 10.

**Remark 12 .**

**Figure 1:**
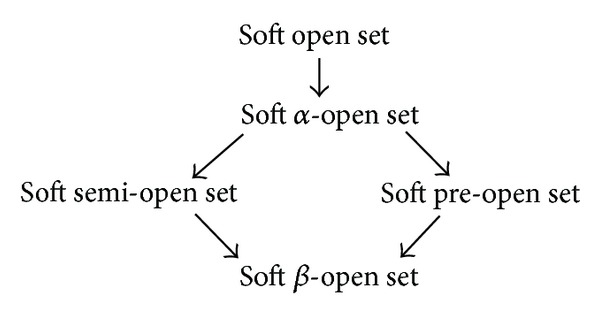


We have following implications; however, the converses of these implications are not true, in general, as shown in Figure 1.

**Example 13 .**

Let *X* = {*x*_1_, *x*_2_, *x*_3_}, *E* = {*e*_1_, *e*_2_, *e*_3_}, and $\tau =\{\Phi ,\tilde{X},(F_{1},E),(F_{2},E),(F_{3},E),...,(F_{15},E)\}$, where (*F*_1_, *E*), (*F*_2_, *E*), (*F*_3_, *E*), ...(*F*_15_, *E*) are soft sets over *X*, defined as follows:

 (*F*_1_, *E*) = {(*e*_1_, {*x*_1_, *x*_2_}), (*e*_2_, {*x*_3_}), (*e*_3_, {*x*_1_, *x*_3_})},
 (*F*_2_, *E*) = {(*e*_1_, {*x*_2_}), (*e*_2_, {*x*_1_, *x*_2_}), (*e*_3_, {*x*_1_, *x*_2_})},
 (*F*_3_, *E*) = {(*e*_1_, {*x*_2_}), (*e*_3_, {*x*_1_})},
 
$\left(F_{4},E)=\{(e_{1},\{x_{1},x_{2}\}),(e_{2},\tilde{X}),(e_{3},\tilde{X})\right\}$,
 (*F*_5_, *E*) = {(*e*_1_, {*x*_3_}), (*e*_2_, {*x*_1_, *x*_3_}), (*e*_3_, {*x*_2_})},
 (*F*_6_, *E*) = {(*e*_2_, {*x*_3_})},
 
$\left(F_{7},E)=\{(e_{1},\tilde{X}),(e_{2},\{x_{1},x_{3}\}),(e_{3},\tilde{X})\right\}$,
 (*F*_8_, *E*) = {(*e*_2_, {*x*_1_}), (*e*_3_, {*x*_2_})},
 
$\left(F_{9},E)=\{(e_{1},\{x_{2},x_{3}\}),(e_{2},\tilde{X}),(e_{3},\{x_{1},x_{2}\})\right\}$,
 (*F*_10_, *E*) = {(*e*_1_, {*x*_2_, *x*_3_}), (*e*_2_, {*x*_1_, *x*_3_}), (*e*_3_, {*x*_1_, *x*_2_})},
 (*F*_11_, *E*) = {(*e*_2_, {*x*_1_, *x*_3_}), (*e*_3_, {*x*_2_})},
 
$\left(F_{12},E)=\{(e_{1},\{x_{2}\}),(e_{2},\tilde{X}),(e_{3},\{x_{1},x_{2}\})\right\}$,
 (*F*_13_, *E*) = {(*e*_1_, {*x*_2_}), (*e*_2_, {*x*_1_}), (*e*_3_, {*x*_1_, *x*_3_})},
 
$\left(F_{14},E)=\{(e_{1},\{x_{1},x_{2}\}),(e_{2},\{x_{1},x_{3}\}),(e_{3},\tilde{X})\right\}$,
 (*F*_15_, *E*) = {(*e*_1_, {*x*_2_}), (*e*_2_, {*x*_3_}), (*e*_3_, {*x*_1_})}.

Then *τ* defines a soft topology on *X*, and thus (*X*, *τ*, *E*) is a soft topological space over *X*. Clearly the soft closed sets are $\tilde{X},\Phi ,{\left(F_{1},E\right)}^{c},{\left(F_{2},E\right)}^{c},{\left(F_{3},E\right)}^{c},...,{\left(F_{15},E\right)}^{c}$.

Then, let us take (*F*, *E*) = {(*e*_1_, {*x*_1_, *x*_3_}), (*e*_2_, {*x*_2_}), (*e*_3_, {*x*_1_, *x*_2_})}; then $\mathrm{int}(F,E)=\tilde{X}$, $\mathrm{int}\left(\mathrm{cl}\left(\mathrm{int}\left(\left(F,E\right)\right)\right)\right)=\tilde{X}$, and so $\left(F,E)\tilde{\subset }\mathrm{int}(\mathrm{cl}(\mathrm{int}((F,E)))\right)$; hence, (*F*, *E*) is soft *α*-open set but not soft open set (since (*F*, *E*) is not soft open set).

Now, let us take (*G*, *E*) = {(*e*_1_, {*x*_2_, *x*_3_}), (*e*_2_, {*x*_3_}), (*e*_3_, {*x*_1_})}; then int⁡((*G*, *E*)) = {(*e*_1_, {*x*_2_}), (*e*_2_, {*x*_3_}), (*e*_3_, {*x*_1_})}, $\mathrm{cl}(\mathrm{int}((G,E)))=\left\{\left(e_{1},\tilde{X}\right),\left(e_{2},\left\{x_{2},x_{3}\right\}\right),\left(e_{2},\left\{x_{1},x_{3}\right\}\right)\right\}$, and so $\left(G,E)\tilde{\subset }\mathrm{cl}(\mathrm{int}((G,E))\right)$; hence, (*G*, *E*) is soft semiopen set but not soft *α*-open set.

Now, let us take (*L*, *E*) = {(*e*_1_, {*x*_2_}), (*e*_2_, {*x*_3_})}; then cl⁡((*L*, *E*)) = (*F*_8_, *E*)^*c*^, int⁡(cl⁡((*L*, *E*))) = {(*e*_1_, {*x*_1_, *x*_2_}), (*e*_2_, {*x*_3_}), (*e*_3_, {*x*_1_, *x*_3_})}, and so $\left(L,E)\tilde{\subset }\mathrm{int}(\mathrm{cl}((L,E))\right)$; hence, (*L*, *E*) is soft preopen set but not soft *α*-open set.

Finally, let us consider $\left(H,E)=\{(e_{1},\tilde{X}),(e_{2},\{x_{2}\}),(e_{3},\{x_{1},x_{3}\})\right\}$ as a soft set in *X*.

Then $\mathrm{cl}(\mathrm{int}(\mathrm{cl}(H,E)))=\tilde{X}$, and so $\left(H,E)\tilde{\subset }\mathrm{cl}(\mathrm{int}(\mathrm{cl}(H,E))\right)$; as a result, (*H*, *E*) is soft *β*-open set, but it is neither soft semiopen set nor soft preopen set.

## 2. Some Properties of Soft *β*-Open Sets and Soft *β*-Closed Sets

Recall that a soft set (*F*, *A*) of a soft topological space (*X*, *τ*, *E*) is said to be soft *β*-open [9] if $\left(F,A)\tilde{\subset }\mathrm{cl}(\mathrm{int}(\mathrm{cl}(F,A))\right)$. The complement of a soft *β*-open set is called soft *β*-closed. Soft *β*-closure and soft *β*-interior of a soft set are defined as follows.

**Definition 14 .**

Let (*X*, *τ*, *E*) be a soft topological space and let (*F*, *A*) be a soft set over *X*.

- Soft *β*-interior of a soft set (*F*, *A*) in *X* is denoted by $s\beta \mathrm{int}((F,A))=\tilde{\cup }\left\{\left(O,A\right):\left(O,A\right)\;\text{is}\;\text{a}\;\text{soft}\;\beta \text{-open}\;\text{set}\;\text{and}\;(O,A)\tilde{\subset }(F,A)\right\}$.
- Soft *β*-closure of a soft set (*F*, *A*) in *X* is denoted by $s\beta \mathrm{cl}((F,A))=\tilde{\cap }\left\{\left(F,E\right):\left(F,E\right)\;\text{is}\;\text{a}\;\text{soft}\;\beta \text{-closed}\;\text{set}\;\text{and}\;(F,A)\tilde{\subset }(F,E)\right\}$.

Clearly *sβ*cl⁡((*F*, *A*)) is the smallest soft *β*-closed set over *X* which contains (*F*, *A*) and *sβ*int⁡((*F*, *A*)) is the largest soft *β*-open set over *X* which is contained in (*F*, *A*).

We will denote the family of all soft *β*-open sets (resp., soft *β*-closed sets) of a soft topological space (*X*, *τ*, *E*) by *SβOS*(*X*, *τ*, *E*) (resp., *SβCS*(*X*, *τ*, *E*)).

**Proposition 15 (see [12]).**

(1) Arbitrary union of soft *β*-open sets is a soft *β*-open set. (2) Arbitrary intersection of soft *β*-closed sets is a soft *β*-closed set.

**Proposition 16 .**

Let (*X*, *τ*, *E*) be a soft topological space and let (*F*, *A*) be a soft set over *X*; then

1. (*F*, *A*) ∈ *SβCS*(*X*, *τ*, *E*)⇔(*F*, *A*) = *sβ*cl⁡((*F*, *A*));
2. (*F*, *A*) ∈ *SβOS*(*X*, *τ*, *E*)⇔(*F*, *A*) = *sβ*int⁡((*F*, *A*)).

**Proof**

(1) Let $(F,A)=s\beta \mathrm{cl}((F,A))=\tilde{\cap }\left\{\left(F,E\right):\left(F,E\right)\;\text{is}\;\text{a}\;\text{soft}\;\beta \text{-closed}\;\text{set}\;\text{and}\;(F,A)\tilde{\subset }(F,E)\right\}$. This shows that $(F,A)\in \left\{\left(F,E\right):\left(F,E\right)\;\text{is}\;\text{a}\;\text{soft}\;\beta \text{-closed}\;\text{set}\;\text{and}\;(F,A)\tilde{\subset }(F,E)\right\}$.

Hence (*F*, *A*) is soft *β*-closed set.

Conversely, let (*F*, *A*) be soft *β*-closed set.

Since $\left(F,A)\tilde{\subset }(F,A\right)$ and (*F*, *A*) is a soft *β*-closed set, $(F,A)\in \left\{\left(F,E\right):\left(F,E\right)\;\text{is}\;\text{a}\;\text{soft}\;\beta \text{-closed}\;\text{set}\;\text{and}\;(F,A)\tilde{\subset }(F,E)\right\}$.

Further, $\left(F,A)\tilde{\subset }(F,E\right)$ for all such (*F*, *E*)'s.

$(F,A)=\tilde{\cap }\left\{\left(F,E\right):\left(F,E\right)\;\text{is}\;\text{a}\;\text{soft}\;\beta \text{-closed}\;\text{set}\;\text{and}\;(F,A)\tilde{\subset }(F,E)\right\}$.

(2) Similar to (1).

**Proposition 17 .**

In a soft space (*X*, *τ*, *E*), the following hold for soft *β*-closure:

1. *sβ*cl⁡(Φ) = Φ.
2. *sβ* *c* *l*((*F*, *A*))* is soft β-closed set in *(*X*, *τ*, *E*)* for each soft subset *(*F*, *A*)* of X*.
3. $s\beta \mathrm{cl}((F,A))\tilde{\subset }s\beta \mathrm{cl}((G,B))$ *, if* $\left(F,A)\tilde{\subset }(G,B\right)$.

**Theorem 18 .**

Let (*X*, *τ*, *E*) be a soft topological space and let (*F*, *A*) and (*G*, *B*) be two soft sets over *X*; then

1. (*sβ*cl⁡((*F*, *A*)))^*c*^ = *sβ*int⁡((*F*, *A*)^*c*^);
2. (*sβ*int⁡((*F*, *A*)))^*c*^ = *sβ*cl⁡((*F*, *A*)^*c*^);
3. $\left(F,A)\tilde{\subset }(G,B)\Rightarrow s\beta \mathrm{int}((F,A))\tilde{\subset }s\beta \mathrm{int}((G,B)\right)$;
4. *sβ*cl⁡(Φ) = Φ and $s\beta \mathrm{cl}(\tilde{X})=\tilde{X}$;
5. *sβ*int⁡(Φ) = Φ and $s\beta \mathrm{int}(\tilde{X})=\tilde{X}$;
6. $s\beta \mathrm{cl}((F,A)\tilde{\cup }(G,B))\tilde{⊃}s\beta \mathrm{cl}((F,A))\tilde{\cup }s\beta \mathrm{cl}((G,B))$;
7. $s\beta \mathrm{int}((F,A)\tilde{\cap }(G,B))\tilde{\subset }s\beta \mathrm{int}((F,A))\tilde{\cap }s\beta \mathrm{int}((G,B))$;
8. *sβ*cl⁡(*sβ*cl⁡((*F*, *A*))) = *sβ*cl⁡((*F*, *A*));
9. *sβ*int⁡(*sβ*int⁡((*F*, *A*))) = *sβ*int⁡((*F*, *A*)).

**Proof**

Let (*F*, *A*) and (*G*, *B*) be two soft sets over *X*.

1. We have ${\left(s\beta \mathrm{cl}((F,A))\right)}^{c}=(\tilde{\cap }\{(F,A):(F,A)\tilde{\subset }(F,A)$ and (*F*, *A*) ∈ *SβCS*(*X*, *τ*, *E*)})^*c*^ $=\tilde{\cup }\{{\left(F,A\right)}^{c}:(F,A)\tilde{\subset }(F,A)$ and (*F*, *A*) ∈ *SβCS*(*X*, *τ*, *E*)}$=\tilde{\cup }\{{\left(F,A\right)}^{c}:{\left(F,A\right)}^{c}\tilde{\subset }{\left(F,A\right)}^{c}$ and (*F*, *A*)^*c*^ ∈ *SβOS*(*X*, *τ*, *E*)} = *sβ*int⁡((*F*, *A*)^*c*^).
2. Similar to (1).
3. Follows from definition.
4. Since Φ and $\tilde{X}$ are soft *β*-closed sets so *sβ*cl⁡(Φ) = Φ and $s\beta \mathrm{cl}(\tilde{X})=\tilde{X}$.
5. Since Φ and $\tilde{X}$ are soft *β*-open sets so *sβ*int⁡(Φ) = Φ and $s\beta \mathrm{int}(\tilde{X})=\tilde{X}$.
6. We have $\left(F,A)\tilde{\subset }((F,A)\tilde{\cup }(G,B)\right)$ and $\left(G,B)\tilde{\subset }((F,A)\tilde{\cup }(G,B)\right)$. Then by Proposition 17 (3), $s\beta \mathrm{cl}((F,A))\tilde{\subset }s\beta \mathrm{cl}((F,A)\tilde{\cup }(G,B))$ and $\mathrm{cl}((G,B))\tilde{\subset }s\beta \mathrm{cl}((F,A)\tilde{\cup }(G,B))$ $\Rightarrow s\beta \mathrm{cl}((G,B))\tilde{\cup }s\beta \mathrm{cl}((F,A))\tilde{\subset }s\beta \mathrm{cl}((F,A)\tilde{\cup }(G,B))$.
7. Similar to (6).
8. Since *sβ*cl⁡((*F*, *A*)) ∈ *SβCS*(*X*, *τ*, *E*), so by Proposition 16 (1), *sβ*cl⁡(*sβ*cl⁡((*F*, *A*))) = *sβ*cl⁡((*F*, *A*)).
9. Since *sβ*int⁡((*F*, *A*)) ∈ *SβOS*(*X*, *τ*, *E*), so by Proposition 16 (2), *sβ*int⁡(*sβ*int⁡((*F*, *A*))) = *sβ*int⁡((*F*, *A*)).

**Theorem 19 .**

For a soft topological space (*X*, *τ*, *E*) the following are valid.

- $\tau \tilde{\subset }S\beta OS(X,\tau ,E)$.
- If (*F*, *A*) is a soft set in *X* and (*G*, *B*) is a soft preopen set in *X* such that $\left(G,B)\tilde{\subset }(F,A)\tilde{\subset }\mathrm{cl}(\mathrm{int}((G,B)\right)$, then (*F*, *A*) is a soft *β*-open set.

**Proof**

(a) The proof is obvious. (b) Since (*G*, *B*) is a soft preopen set we have $\left(G,B)\tilde{\subset }\mathrm{int}(\mathrm{cl}((G,B))\right)$.

Then $\left(F,A)\tilde{\subset }\mathrm{cl}(\mathrm{int}(\mathrm{int}(\mathrm{cl}((G,B)))))=\mathrm{cl}(\mathrm{int}(\mathrm{cl}((G,B))))\tilde{\subset }\mathrm{cl}(\mathrm{int}(\mathrm{cl}((F,A)))\right)$, so (*F*, *A*) is a soft *β*-open set.

**Definition 20 (see [13]).**

Let (*X*, *τ*, *E*) be soft topological space and let *Y* be an ordinary subset of *X*. Then *τ*_*Y*_ = ((*F*, *A*)/*Y* : (*F*, *A*) ∈ *τ*) is a soft topology on *Y* and is called the induced or relative soft topology. The pair (*Y*, *τ*_*Y*_) is called a soft subspace of (*X*, *τ*, *E*): (*Y*, *τ*_*Y*_) is called a soft open/soft closed/soft *β*-open soft subspace if the characteristic function of *Y*, namely, *X*_*Y*_, is soft open/soft closed/soft *β*-open, respectively.

**Theorem 21 .**

Let (*X*, *τ*, *E*) be a soft topological space. Suppose $Z\tilde{\subset }Y\tilde{\subset }X$ and (*Y*, *τ*_*Y*_) is a soft *β*-open soft subspace of (*X*, *τ*, *E*). Then *Z* is soft *β*-open soft subspace in *X* if and only if *Z* is soft *β*-open soft subspace in *Y*.

**Proof**

Suppose that *Z* is soft *β*-open soft subspace in *X*.

Then $X_{Z}\tilde{\subset }\mathrm{cl}(\mathrm{int}(\mathrm{cl}(X_{Z})))$.

But $Z\tilde{\subset }Y$ implies $X_{Z}\tilde{\cap }X_{Y}=X_{Z}$ so that $X_{Z}\tilde{\cap }X_{Y}=X_{Z}\tilde{\subset }\mathrm{cl}(\mathrm{int}(\mathrm{cl}(X_{Z})))=X_{Z}\tilde{\cap }X_{Y}$.

This implies that *X*_*Z*_ is soft *β*-open in *Y*. That is, *Z* is soft *β*-open soft subspace in *Y*.

## 3. Soft *β*-Continuity

**Definition 22 (see [14]).**

Let (*X*, *E*) and (*Y*, *K*) be soft classes. Let *u* : *X* → *Y* and *p* : *E* → *K* be mappings. Then a mapping *f* : (*X*, *E*)→(*Y*, *K*) is defined as follows: for a soft set (*F*, *A*) in (*X*, *E*), (*f*(*F*, *A*), *B*),  *B* = *p*(*A*)⊆*K* is a soft set in (*Y*, *K*) given by *f*(*F*, *A*)(*β*) = *u*(∪*F*(*α*)_*α*∈*p*^−1^(*β*)∩*A*_) for *β* ∈ *K*. (*f*(*F*, *A*), *B*) is called a soft image of a soft set (*F*, *A*). If *B* = *K*, then we will write (*f*(*F*, *A*), *K*) as *f*(*F*, *A*).

**Definition 23 (see [14]).**

Let *f* : (*X*, *E*)→(*Y*, *K*) be a mapping from a soft class (*X*, *E*) to another soft class (*Y*, *K*), and let (*G*, *C*) be a soft set in soft class (*Y*, *K*), where *C*⊆*K*. Let *u* : *X* → *Y* and *p* : *E* → *K* be mappings. Then (*f* ^−1^(*G*, *C*), *D*), *D* = *p* ^−1^(*C*), is a soft set in the soft classes (*X*, *E*), defined as follows: *f* ^−1^(*G*, *C*)(*α*) = *u* ^−1^(*G*(*p*(*α*))) for *α* ∈ *D*⊆*E*.  (*f* ^−1^(*G*, *C*), *D*) is called a soft inverse image of (*G*, *C*). Hereafter we will write (*f* ^−1^(*G*, *C*), *E*) as *f* ^−1^(*G*, *C*).

**Theorem 24 (see [14]).**

Let *f* : (*X*, *E*)→(*Y*, *K*), *u* : *X* → *Y*, and *p* : *E* → *K* be mappings. Then for soft sets (*F*, *A*), (*G*, *B*) and a family of soft sets (*F*_*i*_, *A*_*i*_) in the soft class (*X*, *E*), we have the following:

1. *f*(Φ) = Φ,
2. $f\left(\tilde{X}\right)=\tilde{Y}$,
3. $f\left((F,A)\tilde{\cup }\left(G,B\right)\right)=f(F,A)\tilde{\cup }f\left(G,B\right)$ in general $f\left({\tilde{\cup }}_{i}\left(F_{i},A_{i}\right)\right)={\tilde{\cup }}_{i}f\left(F_{i},A_{i}\right)$,
4. $f\left((F,A)\tilde{\cap }\left(G,B\right)\right)\tilde{\subseteq }f(F,A)\tilde{\cap }f\left(G,B\right)$ in general $f\left({\tilde{\cap }}_{i}\left(F_{i},A_{i}\right)\right)\tilde{\subseteq }{\tilde{\cap }}_{i}f\left(F_{i},A_{i}\right)$,
5. if $(F,A)\tilde{\subseteq }\left(G,B\right)$, then $f(F,A)\tilde{\subseteq }f\left(G,B\right)$,
6. *f* ^−1^(Φ) = Φ,
7. $f^{-1}\left(\tilde{Y}\right)=\tilde{X}$,
8. $f^{-1}\left((F,A)\tilde{\cup }\left(G,B\right)\right)=f^{-1}(F,A)\tilde{\cup }f^{-1}\left(G,B\right)$ in general $f^{-1}\left({\tilde{\cup }}_{i}\left(F_{i},A_{i}\right)\right)={\tilde{\cup }}_{i}f^{-1}\left(F_{i},A_{i}\right)$,
9. $f^{-1}\left((F,A)\tilde{\cap }\left(G,B\right)\right)=f^{-1}(F,A)\tilde{\cap }f^{-1}\left(G,B\right)$ in general $f^{-1}\left({\tilde{\cap }}_{i}\left(F_{i},A_{i}\right)\right)={\tilde{\cap }}_{i}f^{-1}\left(F_{i},A_{i}\right)$,
10. if $(F,A)\tilde{\subseteq }\left(G,B\right)$, then $f^{-1}(F,A)\tilde{\subseteq }f^{-1}\left(G,B\right)$.

Throughout the paper, the spaces *X* and *Y* stand for soft topological spaces with ((*X*, *τ*, *E*) and (*Y*, *v*, *K*)) assumed unless otherwise stated. Moreover, throughout this paper, a soft mapping *f* : *X* → *Y* stands for a mapping, where *f* : (*X*, *τ*, *E*)→(*Y*, *υ*, *K*), *u* : *X* → *Y*, and *p* : *E* → *K* are assumed mappings unless otherwise stated.

**Definition 25 .**

A soft mapping *f* : *X* → *Y* is called soft *β*-continuous [12] (resp., soft *α*-continuous [10], soft precontinuous [10], and soft semicontinuous [15]) if the inverse image of each soft open set in *Y* is soft *β*-open (resp., soft *α*-open, soft preopen, and soft semiopen) set in *X*.

**Remark 26 .**

We have the following implications; however, the converses of these implications are not true, in general, as shown in Figure 2.

**Example 27 .**

Let *X* = {*x*_1_, *x*_2_, *x*_3_}, *Y* = {*y*_1_, *y*_2_, *y*_3_}, *E* = {*e*_1_, *e*_2_, *e*_3_}, and *K* = {*k*_1_, *k*_2_, *k*_3_} and let (*X*, *τ*, *E*) and (*Y*, *υ*, *K*) be soft topological spaces.

Define *u* : *X* → *Y* and *p* : *E* → *K* as

 
*u*(*x*_1_) = {*y*_1_}, *u*(*x*_2_) = {*y*_3_}, *u*(*x*_3_) = {*y*_2_},
 
*p*(*e*_1_) = {*k*_2_}, *p*(*e*_2_) = {*k*_1_}, *p*(*e*_3_) = {*k*_3_}.

Let us consider the soft topology *τ* on *X* given in Example 13; that is,

 
$\tau =\{\Phi ,\tilde{X},(F_{1},E),(F_{2},E),(F_{3},E),...,(F_{15},E)\}$, $\upsilon =\{\Phi ,\tilde{Y},(F,K)\}$;
 (*F*, *K*) = {(*k*_1_, {*y*_1_, *y*_2_}), (*k*_2_, {*y*_3_}), (*k*_3_, {*y*_1_, *y*_3_})} and mapping;
 
*f* : (*X*, *τ*, *E*)→(*Y*, *υ*, *K*) is a soft mapping. Then (*F*, *K*) is a soft open set in *Y*;
 
*f* ^−1^((*F*, *K*)) = {(*e*_1_, {*x*_1_, *x*_3_}), (*e*_2_, {*x*_2_}), (*e*_3_, {*x*_1_, *x*_2_})} is a soft *α*-open set but not soft open set in *X*.

Therefore, *f* is a soft *α*-continuous function but not soft continuous function.

**Example 28 .**

Let *X* = {*x*_1_, *x*_2_, *x*_3_}, *Y* = {*y*_1_, *y*_2_, *y*_3_}, *E* = {*e*_1_, *e*_2_, *e*_3_}, and *K* = {*k*_1_, *k*_2_, *k*_3_} and let (*X*, *τ*, *E*) and (*Y*, *υ*, *K*) be soft topological spaces.

Let us consider the *u* : *X* → *Y* and *p* : *E* → *K* as mapping given in Example 27 and the soft topology *τ* on *X* given in Example 13; that is,

 
$\upsilon =\{\Phi ,\tilde{Y},(G,K)\}$, (*G*, *K*) = {(*k*_1_, {*y*_3_}), (*k*_2_, {*y*_2_})} and mapping;
 
*f* : (*X*, *τ*, *E*)→(*Y*, *υ*, *K*) is a soft mapping. Then (*G*, *K*) is a soft open set in *Y*;
 
*f* ^−1^((*G*, *K*)) = {(*e*_1_, {*x*_2_}), (*e*_2_, {*x*_3_})} is a soft preopen set but not soft *α*-open set in *X*. Thus, *f* is a soft precontinuous function but not soft *α*-continuous function.

**Example 29 .**

Let *X* = {*x*_1_, *x*_2_, *x*_3_}, *Y* = {*y*_1_, *y*_2_, *y*_3_}, *E* = {*e*_1_, *e*_2_, *e*_3_}, and *K* = {*k*_1_, *k*_2_, *k*_3_} and let (*X*, *τ*, *E*) and (*Y*, *υ*, *K*) be soft topological spaces.

Let us consider the *u* : *X* → *Y* and *p* : *E* → *K* as mapping given in Example 27 and the soft topology *τ* on *X* given in Example 13; that is,

 
$\upsilon =\{\Phi ,\tilde{Y},(L,K)\}$, (*L*, *K*) = {(*k*_1_, {*y*_3_, *y*_2_}, (*k*_2_, {*y*_2_}), (*k*_3_, {*y*_1_})} and mapping;
 
*f* : (*X*, *τ*, *E*)→(*Y*, *υ*, *K*) is a soft mapping. Then (*L*, *K*) is a soft open set in *Y*;
 
*f* ^−1^((*L*, *K*)) = {(*e*_1_, {*x*_2_, *x*_3_}), (*e*_2_, {*x*_3_}), (*e*_3_, {*x*_1_})} is a soft semiopen set but not soft *α*-open set in *X*. Hence, *f* is a soft semicontinuous function but not soft *α*-continuous function.

**Example 30 .**

Let *X* = {*x*_1_, *x*_2_, *x*_3_}, *Y* = {*y*_1_, *y*_2_, *y*_3_}, *E* = {*e*_1_, *e*_2_, *e*_3_}, and *K* = {*k*_1_, *k*_2_, *k*_3_} and let (*X*, *τ*, *E*) and (*Y*, *υ*, *K*) be soft topological spaces.

Let us consider the *u* : *X* → *Y* and *p* : *E* → *K* as mapping given in Example 27 and the soft topology *τ* on *X* given in Example 13; that is,

 
$\upsilon =\{\Phi ,\tilde{Y},(H,K)\}$, $\left(H,K)=\{(k_{1},\tilde{Y}),(k_{2},\{y_{3}\}),(k_{3},\{y_{1},y_{2}\})\right\}$ and mapping;
 
*f* : (*X*, *τ*, *E*)→(*Y*, *υ*, *K*) is a soft mapping. Then (*H*, *K*) is a soft open set in *Y*;
 
$f^{-1}((H,K))=\{(e_{1},\tilde{X}),(e_{2},\{x_{2}\}),(e_{3},\{x_{1},x_{3}\})\}$ is a soft *β*-open set, but it is neither soft semiopen set nor soft preopen set in *X*.

Therefore, *f* is a soft *β*-continuous function, but it is neither soft semicontinuous function nor soft precontinuous function.

**Definition 31 (see [12]).**

Let *f* : *X* → *Y* be a function. *f* is called soft *β*-irresolute if the inverse image of soft *β*-open set in *Y* is soft *β*-open in *X*.

**Definition 32 .**

Let *f* : *X* → *Y* be a function. *f* is called soft *β*-open if the image of each soft *β*-open set in *X* is soft *β*-open in *Y*.

**Theorem 33 .**

Let *f* : (*X*, *τ*, *E*)→(*X*, *τ*′, *E*) be a soft continuous and soft open set. Then *f* is soft *β*-open set.

**Proof**

Let (*F*, *A*) be any soft *β*-open set. Then $\left(F,A\right)\tilde{\subset }\mathrm{cl}\left(\mathrm{int}\left(\mathrm{cl}\left(\left(F,A\right)\right)\right)\right)$. Therefore,

 
$f\left(\left(F,A\right)\right)\tilde{\subset }f\left(\mathrm{cl}\left(\mathrm{int}\left(\mathrm{cl}\left(\left(F,A\right)\right)\right)\right)\right)\tilde{\subset }\mathrm{cl}\left(\mathrm{int}\left(\mathrm{cl}\left(f\left(\left(F,A\right)\right)\right)\right)\right)$,
 ⇒*f*((*F*, *A*)) is soft *β*-open.

This shows that *f* is soft *β*-open set.

**Theorem 34 .**

If (*F*, *A*) is soft closed and (*G*, *B*) is soft *β*-open then $\left(F,A)\tilde{\cup }(G,B\right)$ is soft *β*-open.

**Proof**

By hypothesis $\left(G,B)\tilde{\subset }\mathrm{cl}(\mathrm{int}(\mathrm{cl}((G,B)))\right)$. Now

 
$\left(F,A\right)\tilde{\cup }\left(G,B\right)\tilde{\subset }\left(F,A\right)\tilde{\cup }\mathrm{cl}\left(\mathrm{int}\left(\mathrm{cl}\left(\left(G,B\right)\right)\right)\right)\;$,
 
$=\mathrm{cl}(\mathrm{int}(\mathrm{cl}((F,A))))\tilde{\cup }\mathrm{cl}(\mathrm{int}(\mathrm{cl}((G,B))))\tilde{\subset }\mathrm{cl}(\mathrm{int}(\mathrm{cl}((\left(F,A\right)\tilde{\cup }(G,B)))))$.

This shows that $\left(F,A)\tilde{\cup }(G,B\right)$ is soft *β*-open set.

**Theorem 35 .**

Let *f* : (*X*, *τ*, *E*)→(*X*, *τ*′, *E*) be soft continuous and soft open. Then *f* is soft *β*-irresolute.

**Proof**

Let (*F*, *A*) be any soft *β*-open set in *Y*. Then $\left(F,A)\tilde{\subset }\mathrm{cl}(\mathrm{int}(\mathrm{cl}((F,A)))\right)$. Since *f* is soft continuous and soft open it follows that

 
$f^{-1}((F,A))\tilde{\subset }f^{-1}(\mathrm{cl}(\mathrm{int}(\mathrm{cl}((F,A)))))$,
  = cl⁡(*f* ^−1^(int⁡(cl⁡((*F*, *A*))))) $\tilde{\subset }\mathrm{cl}(\mathrm{int}(f^{-1}(\mathrm{cl}((F,A)))))$,
  = cl⁡(int⁡(cl⁡(*f* ^−1^((*F*, *A*))))). This shows that *f* ^−1^((*F*, *A*)) is soft *β*-open.

This shows that *f* is soft *β*-irresolute.

**Proposition 36 .**

A function *f* : *X* → *Y* is soft *β*-irresolute if and only if for every soft *β*-closed set (*F*, *K*) of *Y*, *f* ^−1^((*F*, *K*)) is soft *β*-closed.

**Proposition 37 .**

In a soft topological space (*X*, *τ*, *E*) the following are valid:

- (*F*, *A*)* is soft β*-*open *⇔*sβ*int⁡((*F*, *A*)) = (*F*, *A*).
- (*F*, *A*)* is soft β*-*closed *⇔*sβ*cl⁡((*F*, *A*)) = (*F*, *A*).

**Theorem 38 .**

*f* : (*X*, *τ*, *E*)→(*X*, *τ*′, *E*) is soft *β*-irresolute if and only if for every soft set (*F*, *A*) of *X*, $f(s\beta \mathrm{cl}((F,A)))\tilde{\subset }s\beta \mathrm{cl}(f((F,A)))$.

**Proof**

Suppose that *f* is soft *β*-irresolute. Now *f*(*sβ*cl⁡((*F*, *A*))) is soft *β*-closed set. By hypothesis *f* ^−1^(*sβ*cl⁡(*f*((*F*, *A*)))) is soft *β*-closed set.

And $\left(F,A)\tilde{\subset }f^{-1}(f((F,A)))\tilde{\subset }f^{-1}(s\beta \mathrm{cl}(f((F,A)))\right)$. Hence, by the definition of soft *β*-closure, $s\beta \mathrm{cl}((F,A))\tilde{\subset }f^{-1}(s\beta \mathrm{cl}(f((F,A))))$.

That is $f(s\beta \mathrm{cl}((F,A)))\tilde{\subset }s\beta \mathrm{cl}(f((F,A)))$.

Conversely, suppose that (*F*, *A*) is soft *β*-closed set in *Y*. Now by hypothesis $f(s\beta \mathrm{cl}(f^{-1}((F,A))))\tilde{\subset }s\beta \mathrm{cl}(f(f^{-1}((F,A))))=(F,A)$. This implies $s\beta \mathrm{cl}(f^{-1}((F,A)))\tilde{\subset }f^{-1}((F,A))$ so that *f* ^−1^((*F*, *A*)) = *sβ*cl⁡(*f* ^−1^((*F*, *A*))).

That is *f* ^−1^((*F*, *A*)) is soft *β*-closed set and so *f* is soft *β*-irresolute.

**Theorem 39 .**

*f* : *X* → *Y* is soft *β*-irresolute if and only if for all soft sets (*F*, *K*) of *Y*, $s\beta \mathrm{cl}(f^{-1}((F,K)))\tilde{\subset }f^{-1}(s\beta \mathrm{cl}((F,K)))$.

**Proof**

Suppose *f* is soft *β*-irresolute. Now *sβ*cl⁡((*F*, *K*)) is soft *β*-closed set so that *f* ^−1^(*sβ*cl⁡((*F*, *K*))) is soft *β*-closed set. Since $f^{-1}((F,K))\tilde{\subset }f^{-1}(s\beta \mathrm{cl}((F,K)))$, it follows from the definition of soft *β*-closure that $s\beta \mathrm{cl}(f^{-1}((F,K)))\tilde{\subset }f^{-1}(s\beta \mathrm{cl}((F,K)))$.

Conversely suppose that (*F*, *K*) is soft *β*-closed set in *Y*. Then *sβ*cl⁡((*F*, *K*)) = (*F*, *K*).

Now by hypothesis $s\beta \mathrm{cl}\left(f^{-1}\left(\left(F,K\right)\right)\right)\tilde{\subset }f^{-1}$(*sβ* *c* *l*((*F*, *K*)))  = *f* ^−1^((*F*, *K*)).

Therefore, *sβ*cl⁡(*f* ^−1^((*F*, *K*))) = *f* ^−1^(*sβ*cl⁡((*F*, *K*))) = *f* ^−1^((*F*, *K*)).

Thus, *f* ^−1^((*F*, *K*)) is soft *β*-closed set and so *f* is soft *β*-irresolute.

The following results are easy to establish.

**Proposition 40 .**

Suppose *f* : *X* → *Y* and *g* : *Y* → *Z* are both soft *β*-irresolute. Then *gof* : *X* → *Z* is soft *β*-irresolute.

**Proposition 41 .**

Let *f* : *X* → *Y* be soft continuous and soft open. Then

- *f* *is soft β*-*irresolute;*
- *f* ^−1^(*sβ*cl⁡((*F*, *K*))) = *sβ*cl⁡(*f* ^−1^(((*F*, *K*))))*, with *(*F*, *K*)* being a soft set in Y*.

**Definition 42 .**

Let *X* and *Y* be soft topological spaces. *X* and *Y* are said to be* M*-soft *β*-homeomorphic if and only if there exists *f* : *X* → *Y* such that *f* is 1-1, onto,* M* soft *β*-continuous and soft *β*-open. Such an *f* is called soft *β*-homeomorphism.

**Proposition 43 .**

If *f* : *X* → *Y* is soft *β*-homeomorphism, then *f* ^−1^(*sβ*cl⁡((*F*, *K*))) = *sβ*cl⁡(*f* ^−1^((*F*, *K*))), where (*F*, *K*) is a soft set in *Y*.

**Corollary 44 .**

If *f* : *X* → *Y* is a soft *β*-homeomorphism, then

- *sβ*cl⁡(*f*((*F*, *K*))) = *f*(*sβ*cl⁡((*F*, *K*))),
- *f*(*sβ*int⁡((*G*, *B*))) = *sβ*int⁡(*f*((*G*, *B*))),
- *f* ^−1^(*sβ*int⁡((*F*, *K*))) = *sβ*int⁡(*f* ^−1^((*F*, *K*))).

## 4. Conclusion

In this paper, we introduce the concept of soft *β*-interior and soft *β*-closure of a soft set in topological spaces and study some of their properties. We also introduce the concept of soft *β*-open sets and soft *β*-continuous functions in topological spaces and some of their properties have been established. We hope that the findings in this paper are just the beginning of a new structure and not only will form the theoretical basis for further applications of topology on soft sets but also will lead to the development of information system and various fields in engineering.

## Figures and Tables

**Figure 2:**